# Cebpa is required for haematopoietic stem and progenitor cell generation and maintenance in zebrafish

**DOI:** 10.1098/rsob.240215

**Published:** 2024-11-06

**Authors:** Kemin Chen, Jieyi Wu, Yuxian Zhang, Wei Liu, Xiaohui Chen, Wenqing Zhang, Zhibin Huang

**Affiliations:** ^1^ The Innovation Centre of Ministry of Education for Development and Diseases, School of Medicine, South China University of Technology, Guangzhou, Guangdong 510006, People’s Republic of China; ^2^ Greater Bay Biomedical Innocenter, Shenzhen Bay Laboratory, Shenzhen, Guangdong 518055, People’s Republic of China

**Keywords:** Cebpa, haematopoiesis, haematopoietic stem and progenitor cell, zebrafish

## Abstract

The CCAAT enhancer binding protein alpha (CEBPA) is crucial for myeloid differentiation and the balance of haematopoietic stem and progenitor cell (HSPC) quiescence and self-renewal, and its dysfunction can drive leukemogenesis. However, its role in HSPC generation has not been fully elucidated. Here, we utilized various zebrafish *cebpa* mutants to investigate the function of Cebpa in the HSPC compartment. Co-localization analysis showed that *cebpa* expression is enriched in nascent HSPCs. Complete loss of Cebpa function resulted in a significant reduction in early HSPC generation and the overall HSPC pool during embryonic haematopoiesis. Interestingly, while myeloid differentiation was impaired in *cebpa* N-terminal mutants expressing the truncated zP30 protein, the number of HSPCs was not affected, indicating a redundant role of Cebpa P42 and P30 isoforms in HSPC development. Additionally, epistasis experiments confirmed that Cebpa functions downstream of Runx1 to regulate HSPC emergence. Our findings uncover a novel role of Cebpa isoforms in HSPC generation and maintenance, and provide new insights into HSPC development.

## 1. Introduction

Haematopoietic stem and progenitor cells (HSPCs) are generated through the endothelial-to-haematopoietic transition (EHT), a highly conserved process across zebrafish and mammals [1,2]. In zebrafish, a model organism ideal for studying haematopoiesis, HSPCs initially emerge from the haemogenic endothelium in the ventral wall of the dorsal aorta (VDA) and the posterior blood island (PBI) around 30 h post-fertilization (hpf) [1,3,4]. These VDA-originated HSPCs subsequently migrate and expand to the caudal haematopoietic tissue (CHT) by 3 days post-fertilization (dpf), eventually colonizing major haematopoietic organs such as the thymus and kidney [5,6]. This process is analogous to the generation of mammalian HSPCs in the aorta–gonad–mesonephros (AGM) region and their migration to the fetal liver and bone marrow for sustained haematopoiesis [2,6,7].

Transcription factors play a pivotal role in regulating HSPC development. Essential factors such as SCL, GATA2 and RUNX1 are crucial for the generation and viability of HSPCs in both zebrafish and mammals [8–11]. Similarly, C-MYB and ERG are highly expressed in haematopoietic stem cells (HSCs), and their dysfunction leads to severe reductions in HSPC numbers [12–15]. MYC and FOXO3 are necessary for HSPC survival and proliferation, while NRF2 maintains HSC stemness [16–18]. FOXC1 has been identified as a protector of HSPCs by forming haematopoietic microenvironments [19]. Despite these discoveries, the molecular regulatory mechanisms underlying HSPC generation and maintenance are not yet fully understood.

CCAAT enhancer binding protein alpha (CEBPA) is a well-established regulator of myeloid cell differentiation [20,21]. The full-length CEBPA protein contains N-terminal transactivation domains (TADs) for transcriptional regulation [22] and a C-terminal basic region and leucine zipper (bZIP) domain for DNA binding and dimerization [23]. CEBPA induces common myeloid progenitors (CMPs) to generate granulocyte-macrophage progenitors (GMPs) through myeloid-specific gene induction and cell cycle control [21,24,25], with high CEBPA expression favouring neutrophil production over macrophages [26]. Dysfunction of CEBPA is frequently observed in myeloid disorders, particularly acute myeloid leukaemia (AML) [27,28], where approximately 10% of de novo AML patients harbour CEBPA mutations [29,30]. These mutations often include the N-terminal frameshift mutation that produces a truncated P30 isoform, which lacks the first TAD domain and promotes leukaemic cell proliferation [30,31].

Previous studies have shown that CEBPA is expressed in a small population of bone marrow HSPCs, as demonstrated by single-cell RNA sequencing and YFP-tagged reporter mice [32,33]. Genome-wide association studies have identified *CEBPA* variants that probably impact CD34^+^ HSPC levels [34]. In mouse models, CEBPA deficiency enhances HSC self-renewal and reconstitution capacity via BMI1 activation [35]. Consistently, HSC expansion is observed in *Cebpa* knock-in mice with C-terminal in-frame mutations, facilitating malignant AML [36]. These findings suggest that CEBPA is essential for maintaining HSPCs in a quiescent state. However, other evidence indicates that CEBPA knockout mice exhibit dramatically reduced numbers and weakened repopulating ability of long-term HSCs due to self-renewal loss and excessive apoptosis [37]. This is further supported by maintenance defects in HSPCs caused by reduced CEBPA expression in enhancer deletion models [38]. Variations in the timing and methods of CEBPA knockout/knockdown and *ex vivo* HSPC examination may contribute to inconsistent results regarding CEBPA’s role in HSPCs. Therefore, there is a need for *in vivo* models to elucidate the functions of CEBPA and its isoforms in HSPC generation and maintenance.

In our study, we identified enriched *cebpa* expression in haemogenic endothelium and found that HSPC generation was disturbed in genetic *cebpa*-null mutants, which ensured the absence of Cebpa production. Excessive apoptosis compromised the HSPC pool and multi-lineage cells, except for erythrocytes, in *cebpa*-null but not in truncated zP30-producing embryos. Additionally, *cebpa* was verified to act downstream of Runx1 to promote HSPC generation. Our work illustrates the crucial role of *cebpa* in safeguarding HSPC development and provides new insights into the role of Cebpa isoforms in HSPC generation and maintenance.

## 2. Material and methods

### 2.1. Zebrafish husbandry

All embryos and adult zebrafish were maintained according to standard protocols [39]. Embryos were raised in egg water (system water with methylene blue) at 28.5℃ until 5 dpf. For experimental use, egg water containing 0.003% PTU (P7629, Sigma-Aldrich) was replaced before 24 hpf to inhibit pigmentation. Strains including AB, *Tg(flk1:GFP*) [40], *Tg(cmyb:GFP*) [41], *Tg(runx1:en-GFP*) [42], *Tg(cd41:eGFP*) [43], *Tg(hsp70:myc-runx1*) [44], *cebpa^hkz7^* [45], *cebpa^TKO^*, *cebpa^Nm^* and *runx1^W84X^* [46] were used in this study.

### 2.2. Generation and identification of *cebpa^TKO^* zebrafish

The *cebpa* mutated zebrafish line with a long fragment deletion was created using CRISPR/Cas9 gene editing technology as previously described [47]. Two sgRNAs targeting the 5'-UTR (target1: 5'-GGCTGCAAGTTCTCTGGAC−3') and 3' coding region (target2: 5'-GGAACACCTCACGCGAGAAC−3') of the *cebpa* gene were designed, synthesized *in vitro* using T7 polymerase (EP0111, Thermo Fisher Scientific) and purified with TRIzol (15596026CN, Invitrogen). After efficiency validation through T7E1 digestion (M0302S, New England Biolabs), a mixture of sgRNAs (150 ng μl^−1^) and Cas9 protein (500 ng μl^−1^, M0646M, New England Biolabs) was micro-injected into one-cell stage wild-type embryos to establish F0 founders and analyse mutation sites in F1 adult fish. The stable line of *cebpa^TKO^* mutants was identified by PCR with the following primers: FP (5'-GTTCTAGGTCTATCAGTGCGTCC−3'), RP (5'-CAGAACGTGTCAGCGGTTATC−3').

### 2.3. Whole mount *in situ* hybridization

Embryos were collected and fixed at designated stages. Whole mount *in situ* hybridization (WISH) for *cebpa*, *cmyb*, *runx1*, *flk1*, *lyz*, *mfap4*, *coro1a*, *βe1*, *gata1*, *pu.1, gata2b* and *rag1* was performed according to previously described procedures [48]. All samples were imaged using a Zeiss Axio Imager.C1 or Zeiss Axio Zoom v. 16 microscope, and each embryo was lysed to obtain genomic DNA for further genotyping by PCR.

### 2.4. Fluorescence *in situ* hybridization, immunofluorescence staining and imaging

For co-staining analysis, transgenic positive embryos at specific stages were collected and stained with standard FISH assays followed by antibody staining, as previously described [49]. Briefly, dig-RNA-labelled *cebpa* and *runx1*/*cmyb* probes were incubated overnight, then primary colouring was performed using HRP-conjugated anti-digoxigenin antibody (11207733910, Roche, 1:3000) and the TSA-Cy3 fluorescence system (NEL744001KT, Akoya Biosciences). Embryos were further co-stained with goat anti-GFP antibody (ab6658, Abcam, 1:400) and Alexa Fluor 488 anti-goat antibody (A21206, Invitrogen, 1:400) for co-localization observation. Fluorescent images were captured using a Zeiss LSM800 confocal microscope with 10× or 20× objectives.

### 2.5. Bromodeoxyuridine labelling and antibody staining

Embryos at 3 dpf from intercrossed *Tg(cd41:eGFP*)^+^;*cebpa^TKO/+^* adults were continuously incubated with 10 mM bromodeoxyuridine (BrdU) dissolved in egg water PTU for 3 h and then recovered for 30 min before fixation. Further staining experiments were performed as previously reported [50]. Genomic identification was completed before 2N HCl treatment. The *cebpa* mutants and siblings were separately collected for antibody incubation and colouring. Antibodies used included mouse anti-BrdU (1117037600, Roche, 1:200), goat anti-GFP (ab6658, Abcam, 1:400), Alexa Fluor 555 anti-mouse (A31570, Invitrogen, 1:400) and Alexa Fluor 488 anti-goat (A21206, Invitrogen, 1:400). Images were captured by confocal microscope, and percentages of double positive cells in Cd41:eGFP^low^ HSPCs were quantified and compared between siblings and mutants.

### 2.6. TUNEL assay and antibody staining

The TUNEL-based cell death detection was conducted using the *in situ* Cell Death Detection Kit TMR red (12156792910, Roche) [50]. Cd41:eGFP^+^ embryos at 3 dpf with or without homozygous *cebpa* mutations were fixed in 4% PFA, gradually dehydrated with methanol and frozen at −20℃ for at least 2 hours. The embryos were rehydrated, digested with proteinase K (10 μg ml^−1^ for 10 min, EO0491, Thermo Scientific) and permeabilized with an acetone-ethanol mixture (1:2) for 7 min at −20℃ before incubation with TMR red labelling solution at 37℃ overnight. After TUNEL staining, the embryos were co-stained with goat anti-GFP (ab6658, Abcam, 1:400) and Alexa Fluor 488 anti-goat (A21206, Invitrogen, 1:400). Images were taken with a confocal microscope, followed by genotyping, and percentages of TUNEL-positive apoptotic cells in Cd41:eGFP^low^ HSPCs were analysed.

### 2.7. RNA extraction, cDNA construction, mRNA synthesis and micro-injection

Twenty to thirty wild-type or *cebpa^Nm^* embryos staged at 36 hpf were collected for total RNA extraction using TRIzol reagent (15596018CN, Invitrogen). cDNA was synthesized with One-step RT-gDNA Digestion SuperMix (11 151ES60, Yeasen) following the manufacturer’s protocol. *cebpa^WT^*, *cebpa^Nm^* and zP30 coding sequences were amplified from cDNA templates by high fidelity PCR and assembled into PCS2-P2A-GFP constructs using the ClonExpress Ultra One Step Cloning Kit (C115, Vazyme).

Various mRNAs were *in vitro* transcribed and purified with the sp6 mMESSAGE synthesis Kit (AM1340, Invitrogen). These mRNAs were micro-injected into one-cell stage *cebpa^TKO^* or sibling embryos at the following concentrations: 120 ng μl^−1^ (zP30 mRNA), 250 ng μl^−1^ (*cebpa^WT^* mRNA) and 500 ng μl^−1^ (*cebpa^Nm^* mRNA).

### 2.8. Inhibitor treatment

PP242 (AM1340, MCE) was dissolved in DMSO to a 50 mM concentration as a stock solution and stored at −80℃. Progeny of intercrossed *cebpa^Nm^*^/+^ adults were collected, and membranes were removed at 4 hpf. Embryos were soaked in 5 μM PP242 solution diluted with egg water PTU until 36 hpf or 3 dpf for phenotype analysis. Control embryos were treated with DMSO under the same conditions. For longer treatments until 3 dpf, the egg water was replaced with freshly diluted inhibitor solution at 36 hpf.

### 2.9. Heat shock treatment

For *runx1* overexpression, progenies of Tg(*hsp70:myc-runx1*) transgenic zebrafish were subjected to two heat shock procedures via water bath at 39.5℃ for 1 h at 11 hpf and 24 hpf.

### 2.10. Statistical analysis

All data were analysed using GraphPad Prism 9 software. Unpaired Student’s *t*‐test and Mann–Whitney test (non-parametric) were used for comparisons between two groups, and two-tailed *p*-values were calculated. One-way ANOVA with Tukey’s adjustment or Kruskal–Wallis test (non-parametric) with Dunn’s adjustment was used for multiple group comparisons. Data were presented as mean ± standard deviation (s.d.), and *p *< 0.05 was considered statistically significant.

## 3. Results

### 3.1. *cebpa* is expressed in haemogenic endothelium and nascent HSPCs in embryonic zebrafish

**Figure 1.**
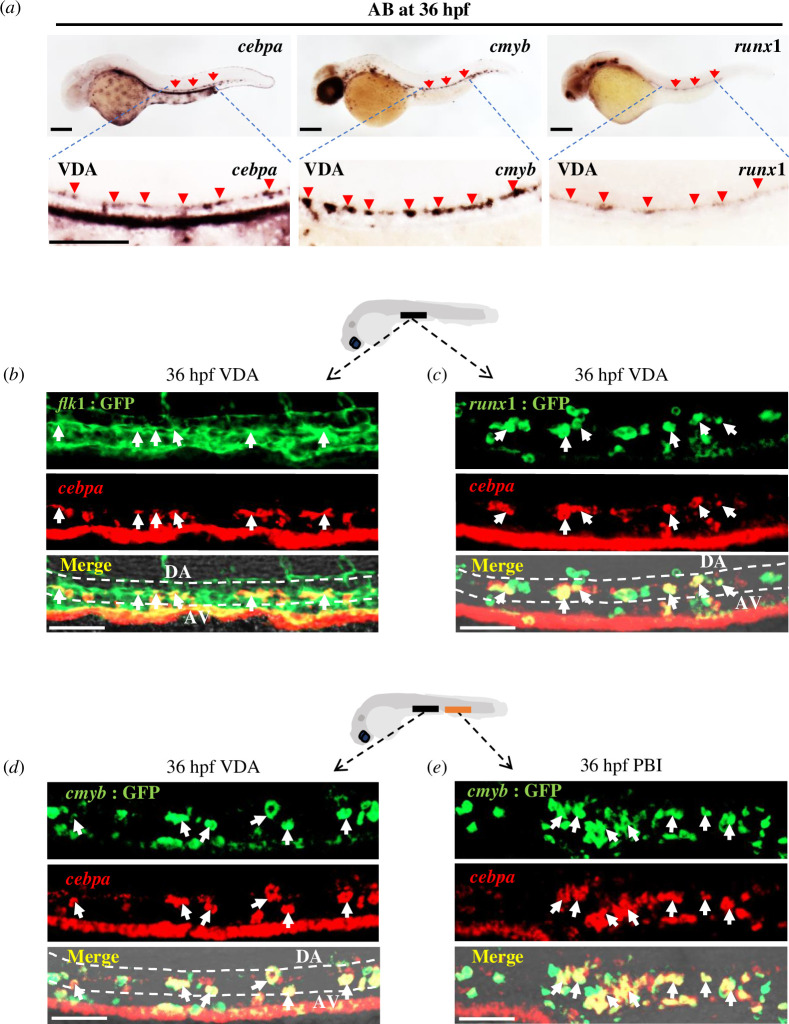
Enriched expression of *cebpa* in zebrafish haemogenic endothelium and HSPCs in early haematopoiesis. (*a*) WISH of *cebpa* in wild-type (AB strain) embryos showed similar expression patterns in the VDA as HSPC markers *cmyb* and *runx1* at 36 hpf. Enlarged details of signals in the VDA indicated by red arrowheads were shown in lower panel images. Scale bars: 200 μm. *n* ≥ 10. (*b, c*) Co-staining of *cebpa* by FISH (red) and anti-GFP staining (green) in *Tg(flk1:GFP)* embryos (*b*) and *Tg(runx1:en-GFP)* embryos (*c*) at 36 hpf. The black line in the top diagram showed the observation region (VDA) of 36 hpf zebrafish embryos. White arrowheads indicated co-expression of *cebpa* in GFP^+^ HE cells or HSPCs restricted to the VDA (shown by white dashed lines). (*d, e*) *cebpa* FISH followed by anti-GFP staining in the CHT of *Tg(cmyb:GFP)* embryos at 36 hpf. The black and orange lines in top diagram showed the observation region of VDA and PBI of 36 hpf embryos. White arrows indicated co-localization of *cebpa* and nascent *cmyb*:GFP*^+^*HSPCs in both VDA (*d*) and PBI (*e*). DA, dorsal aorta; AV, axial vein. Scale bars: 50 μm. *n* ≥ 12.

A minor proportion of *CEBPA*^+^ haematopoietic progenitor cells have been identified as long-term haematopoietic stem cells (LT-HSCs) or lymphoid-primed multipotent progenitors (LMPPs) during early haematopoiesis in mammals [32,51]. In zebrafish, *cebpa* was first detected at 5.3 hpf with subsequent expression in primitive haematopoietic progenitors [52]. However, whether *cebpa* is expressed in definitive HSPCs in zebrafish remains unknown. Through WISH with a *cebpa* probe, we noticed that high *cebpa* expression coincided with *cmyb* and *runx1*, two representative HSPC markers, in the VDA region at 30 hpf (electronic supplementary material, figure S1) and 36 hpf; figure 1*a*), which correspond to the time and location where definitive HSPCs are continuously generated from haemogenic endothelium (HE) cells [1]. To determine whether the *cebpa*^+^ cells located in the VDA were HE cells or newly formed HSPCs, fluorescence *in situ* hybridization was conducted at 36 hpf in various transgenic embryos. Co-staining analysis revealed that *cebpa* was indeed expressed in *flk1*^+^ endothelial cells situated at the floor of the dorsal aorta (figure 1*b*). Additionally, a significant population of *cebpa*^+^ cells exhibited extensive co-expression with *runx1* and *cmyb* in the VDA and PBI of 36 hpf embryos (figure 1*c–e*). Collectively, these observations demonstrated that *cebpa* expression is enriched in both HSPCs and early HE cells in zebrafish, suggesting a potential role for Cebpa in the regulation of HSPC development.

### 3.2. CRISPR/Cas9 mediated total Cebpa knockout in zebrafish with a long fragment deletion

To investigate the role of *cebpa* in HSPC development, we monitored *cmyb^+^* cells using WISH in *cebpa^hkz7^* embryos, a Cebpa-dysfunctional zebrafish line created by our team. This line features a 10 kb insertion disrupting the C-terminal bZIP domain [45]. Surprisingly, *cmyb* expression at 36 hpf and 3 dpf showed no significant difference in the VDA or the CHT between siblings and mutants (electronic supplementary material, figure S2*a*,*b*), suggesting that HSPCs were normally generated and maintained in *cebpa^hkz7^* mutant. This conclusion was further supported by the unaffected expression of the definitive lymphoid marker *rag1* in *cebpa^hkz7^* mutants (electronic supplementary material, figure S2*c*,*d*).

**Figure 2.**
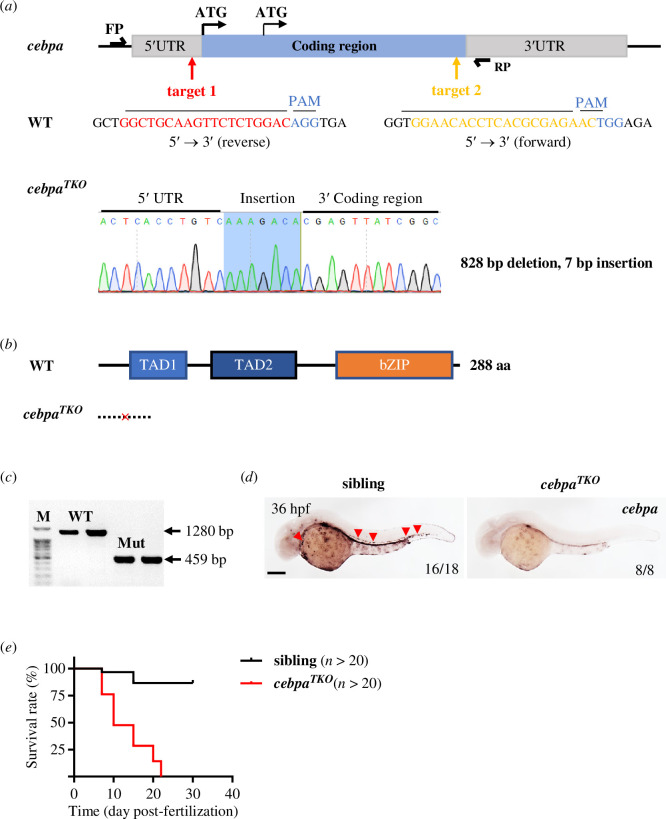
Generation and characterization of *cebpa^TKO^* zebrafish by CRISPR/Cas9 technology. (*a*) Two sgRNA targets were designed for multi-site cleavage of zebrafish *cebpa* gene. Sequences marked in red and orange indicate target 1 located in the reverse strand of the 5′-UTR and target 2 in the forward strand of the 3′ coding region, respectively. Nucleotides of PAM were shown in blue. UTR, untranslated region; PAM, protospacer adjacent motif. Compared to the wild-type (WT) genome, the *cebpa^TKO^* mutant harboured an 828 bp deletion from the 5′-UTR to the end of the Cebpa coding region with an additional 7 bp insertion, resulting in the absence of translational initiation sites. The blue box showed the specific nucleotides produced by insertion. (*b*) The mutated *cebpa* genome was predicted to cause a failure of Cebpa protein production, while wild-type *cebpa* could encode the dominant full-length Cebpa (288 aa) involving two TAD domains and the C-terminal bZIP domain. TAD, transactivation domain; bZIP, basic region and leucine zipper; aa, amino acids. (*c*) The fragment size of PCR products from the *cebpa* mutant genome (459 bp) was approximately 800 bp shorter than that of wild-type (1280 bp) with primers outside mutation sites. M, 100 bp plus DNA marker. (*d*) *cebpa* RNA expression in *cebpa^TKO^* embryos was almost completely lost at 36 hpf by WISH. Red arrowheads showed abundant *cebpa* expression at haematopoietic tissue in sibling embryos. *n* ≥ 8 in each group. Scale bar: 200 μm. (*e*) Survival curve of sibling and *cebpa^TKO^* embryos (*n* > 20 in each group).

Considering that *cebpa^hkz7^* mutants could still encode a truncated protein with approximately 80% coverage of Cebpa, potentially retaining partial function, we generated a novel *cebpa*-mutant line with complete loss of function to enable more accurate phenotypic analysis. Using the CRISPR/Cas9 system, we designed two highly efficient sgRNAs targeting the 5′-UTR and the end of the coding region of the zebrafish *cebpa* gene. These sgRNAs were co-injected with Cas9 protein into one-cell stage wild-type embryos to produce stable lines with a long fragment deletion (figure 2*a*). We successfully screened a heritable genetic *cebpa*-null allele, named *cebpa^TKO^*, which harboured an 828 bp deletion along with a concomitant 7 bp insertion, leading to disabled Cebpa translation due to the absence of all ATGs in the upstream open reading frame (uORF) and the main open reading frame (ORF) (figure 2*a,b*). Genotyping identified this allele by the presence of a much shorter PCR fragment in homozygous mutants, and *cebpa* mRNA expression was rarely detected in mutant embryos through *cebpa* WISH (figure 2*c,d*). Survival analysis indicated a short life expectancy of approximately 22 days (figure 2*e*), corresponding to the embryonic lethality reported in *cebpa*-mutated mice and zebrafish [45,53]. This novel *cebpa*-null mutant line provides a robust model for studying the complete loss of Cebpa function and its effects on HSPC development in zebrafish.

### 3.3. HSPC generation process was disturbed in the total absence of Cebpa

To assess the impact of Cebpa loss on blood cell generation during developmental haematopoiesis, we examined the expression of related gene markers. Initially, comparable *gata1* expression in the intermediate cell mass (ICM) between siblings and *cebpa^TKO^* homozygous embryos at 20 hpf suggested normal production of primitive erythrocytes (electronic supplementary material, figure S3*a*). However, markedly decreased *pu.1* expression in *cebpa^TKO^* mutants indicated a primitive myelopoietic defect, characterized by migration defects of normally initiated progenitors from the rostral blood island (RBI) to the yolk sac as reported (electronic supplementary material, figure S3*b*) [45].

**Figure 3.**
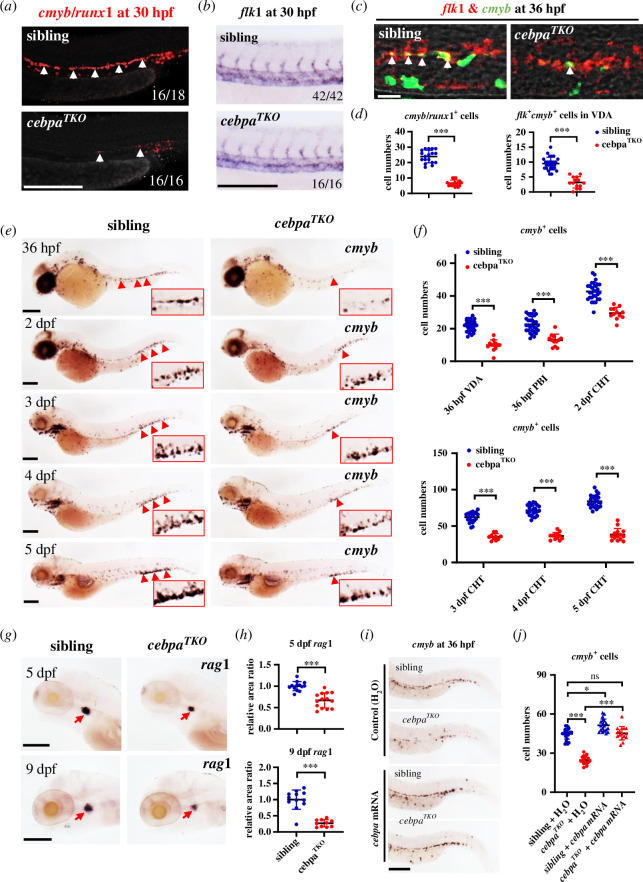
Complete loss of Cebpa leads to reduced HSPC generation and lymphopoiesis in zebrafish**.** (*a*) Decreased *cmyb*/*runx1* expression in *cebpa^TKO^* mutants at 30 hpf by FISH with the mixed probe of *cmyb* and *runx1*. White arrowheads indicated positive signals in the VDA region. Scale bar: 200 μm. (*b*) Comparable *flk1* expression in sibling and *cebpa^TKO^* mutants at 30 hpf by WISH. The numbers in the lower right corner indicated the ratio of embryos with representative expressing patterns in whole embryos with identical genotypes. Scale bar: 200 μm. (*c*) Co-staining analysis showed decreased *flk1*^+^*cmyb*^+^ cells in *cebpa^TKO^* mutants at 36 hpf. Red fluorescence indicated vascular endothelium and green fluorescence signed nascent HSPCs. White arrowheads indicated double positive HE cells in the VDA region. Scale bar: 20 μm. (*d*) Quantification and analysis of *cmyb/runx1^+^* or *flk1*^+^*cmyb*^+^ cells in VDA of sibling and *cebpa^TKO^* embryos (mean ± s.d.; *n* ≥ 14; Student’s *t*‐test, ***p < 0.001). (*e*) *cmyb* WISH showed a sustainable decrease of *cmyb^+^* + from 36 hpf in the VDA to later 2–5 dpf in the CHT of *cebpa^TKO^* mutants. Enlarged signal details were shown in red boxes. Scale bars: 200 μm. (*f*) Quantification of *cmyb*^+^ cell numbers in the VDA or CHT region at corresponding stages of sibling and *cebpa* mutants (mean ± s.d.; *n* ≥ 10; Student’s *t*‐test and non-parametric Mann–Whitney test, ***p < 0.001). (*g*) WISH of *rag1* in the thymus of sibling and *cebpa^TKO^* mutants at 5 dpf (left) and 9 dpf (right). *cebpa^TKO^* mutant showed reduced *rag1* (indicated by red arrows) expression. Scale bars: 200 μm. (*h*) Comparison of relative *rag1*^+^ signal area in the thymus (*rag1*-expressing area in each sample was measured by image J and all data divided by average area value in sibling as a reference) between sibling and mutants (mean ± s.d.; *n* ≥ 10; Student’s *t*‐test and non-parametric Mann–Whitney test, ***p < 0.001). (*i*) Re-introduction of *cebpa* expression by *cebpa* mRNA injection could restore *cmyb*^+^ HSPCs in *cebpa^TKO^* embryos. Scale bar: 200 μm. (*j*) Quantification of *cmyb*^+^ cell numbers in the tail of each group of embryos with *cebpa* mRNA or H_2_O (control) injection (mean ± s.d.; *n *≥11; one way ANOVA, ns *p* > 0.05, *p < 0.05, ***p < 0.001).

We then monitored definitive haematopoiesis. Unlike previous observation in *cebpa*-mutated zebrafish, *cmyb* and *runx1* expression at 30 hpf showed a notable defect in the VDA and PBI of *cebpa^TKO^* embryos compared to sibling controls (figure 3*a*,*d*; electronic supplementary material, figure S4*a*,*c*). However, *gata2b*, which is enriched in early HE cells prior to *runx1* [54], and *flk1* expression in mutants was unaffected (figure 3*b*; electronic supplementary material, figure S4*b,d*), indicating that HSPC emergence was impaired while vasculogenesis and HE specification remained normal in response to *cebpa* deficiency. Moreover, reduced *flk1*^+^*cmyb*^+^ cells in the aortic floor were also observed in *cebpa*-null mutants (figure 3*c,d*), and *cmyb*^+^ cells were significantly decreased in the VDA at 36 hpf in *cebpa^TKO^* mutants, followed by a constant decline in the CHT from 2 dpf to 5 dpf (figure 3*e,f*). Collectively, these results suggested that Cebpa is necessary for HSPC generation in zebrafish.

To assess the ability of HSPCs to produce all blood cell lineages, we evaluated multi-lineage cell development using WISH for specific markers. The results showed that *rag1* expression in the thymus of 5 dpf *cebpa^TKO^* mutants was significantly lower than in sibling embryos, with the disparity becoming more pronounced by 9 dpf, indicating disrupted lymphocyte development in *cebpa*-null mutants (figure 3*g,h*). Myelopoiesis defects were also evident, with a lack of mature myelocyte staining (Sudan black B staining) or markers (*lyz*, *mfap4*), and a substantial reduction of *coro1a*^+^ myeloid progenitor cells in *cebpa^TKO^* mutants (electronic supplementary material, figure S5*a*,*b*), while erythrocytes marked by *βe1* remained unaffected (electronic supplementary material, figure S5*c*).

These observations collectively indicated that the loss of Cebpa function compromised HSPC number and differentiation ability during embryonic haematopoiesis. To confirm *cebpa* as the causative gene for the HSPC defect in *cebpa^TKO^* mutants, we conducted a rescue assay by overexpressing *in vitro* synthesized wild-type zebrafish *cebpa* mRNA at the one-cell stage. WISH examination at 36 hpf showed that *cebpa* mRNA robustly restored *cmyb*^+^ cells in homozygous mutants and increased *cmyb* expression in sibling embryos as well (figure 3*i,j*).

In summary, these findings underscore the essential role of *cebpa* in the generation and subsequent lineage differentiation of HSPCs in the early definitive haematopoietic wave.

### 3.4. HSPCs undergo excessive apoptosis in *cebpa^TKO^* mutants

**Figure 4.**
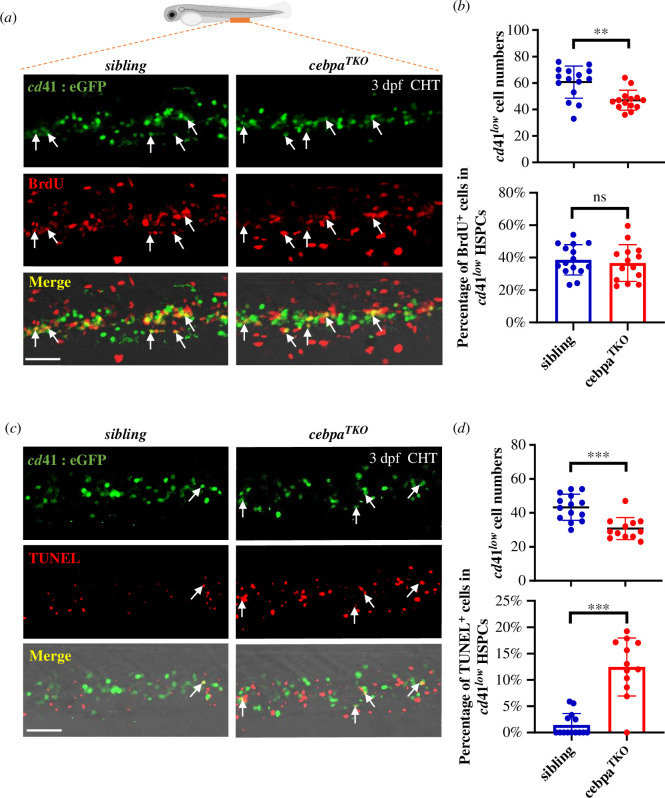
Enhanced apoptosis of HSPCs in the CHT of *cebpa^TKO^* mutant**.** (*a*) Unaffected HSPC proliferation in *cebpa^TKO^* mutants. Double immunofluorescence staining of *cd41*:eGFP (green) and BrdU-labelling (red) cells in sibling and *cebpa^TKO^* embryos at 3 dpf. White arrows indicated BrdU positive proliferating *cd41*:eGFP^low^ HSPCs in the CHT. (*b*) Quantification of cell numbers of single positive *cd41*:eGFP^low^ HSPCs (upper panel) and percentage of co-stained BrdU^+^ HSPCs (lower panel) in sibling and mutants (mean ± s.d.; *n* ≥ 14; Student’s *t*‐test, ns *p* > 0.05, **p < 0.01). (*c*) Increased apoptotic cell death of HSPC (labelled by *cd41*:eGFP^low^ in green) in *cebpa^TKO^* mutants was demonstrated by TUNEL (red) assay. White arrowheads indicated double positive (TUNEL^+^; *cd41*:eGFP^low^) HSPCs suffering apoptosis in the CHT at 3dpf. (*d*) Quantification of *cd41*:eGFP^low^ HSPC numbers (upper panel) and apoptotic cell percentage (lower panel) presented in 3 dpf sibling and mutants (mean ± s.d.; *n* ≥ 12; non-parametric Mann–Whitney test, ***p < 0.001). Scale bars: 50 μm.

Since nascent HSPCs emerging from HE cells are quantitatively restricted, their maintenance and expansion are indispensable for establishing a progenitor cell pool in the CHT region at 2 dpf and beyond [55,56]. Therefore, in addition to the impediment in generation, we investigated whether any cellular alterations contribute to the diminished HSPCs in the CHT of *cebpa^TKO^* mutants. To address this question, we evaluated the cell status regarding proliferation and apoptosis of HSPCs labelled by Cd41 in progeny from inter-crossed *cebpa^TKO^* heterozygous zebrafish with a transgenic positive background. After incubating with BrdU, followed by the co-staining analysis of 3 dpf embryos, *cebpa^TKO^* mutants showed a markedly decreased number of Cd41^low^ HSPCs but an unaffected BrdU^+^ proliferating cell percentage in the CHT region (figure 4*a,b*). This suggested that *cebpa* deficiency does not influence HSPC proliferation.

Similarly, we applied the transferase-mediated dUTP nick-end labelling (TUNEL) assay to evaluate cell death in *cebpa^TKO^* mutants. A significantly elevated percentage of Cd41^low^ HSPCs simultaneously marked by TUNEL was observed in the mutants (figure 4*c,d*), indicating excessive apoptotic cell death of *cebpa*-null HSPCs. Consistently, increased apoptosis of *cmyb*^+^ HSPCs was presented in *cebpa^TKO^* mutant embryos at an early stage of 30 hpf (electronic supplementary material, figure S6*a–c*). These results demonstrated that the reduction of HSPCs in response to Cebpa loss is primarily due to increased apoptosis, emphasizing the essential protective role of zebrafish Cebpa in HSPC maintenance, consistent with findings reported in mice [37,38].

### 3.5. Truncated Cebpa zP30 produced by *cebpa^Nm^* mutation compensates for zP42 Cebpa function in HSPC development

Alternative translation of mRNA encoding CEBPA from specific translation initiation sites has been observed in both mammalian [57,58] and zebrafish [52]. Full-length P42, the predominant isoform, plays multifaceted roles in haematopoiesis, while truncated P30, lacking the first TAD domain, is unexpectedly increased in most AML patients with classical N-terminal frameshift *CEBPA* mutations [31]. Considerable attention has been given to understanding the functional properties of the dominant-negative P30 in myelopoiesis, particularly in the context of leukemogenesis. However, whether CEBPA P30 plays a role in HSPC development remains unclear. In previous research, we obtained a unique *cebpa*-mutant zebrafish [45], referred to as *cebpa^Nm^* here, characterized by a 7 bp N-terminal frameshift deletion identified before the predicted zP30 initiation sites (electronic supplementary material, figure S7*a*). The *cebpa^Nm^* mutants displayed a slight decrease in *cebpa* expression and survived for more than 40 days (electronic supplementary material, figure S7*b*–*d*), more than double the lifespan of *cebpa^TKO^* mutants, suggesting potential zP30 generation in *cebpa^Nm^* mutants.

**Figure 5.**
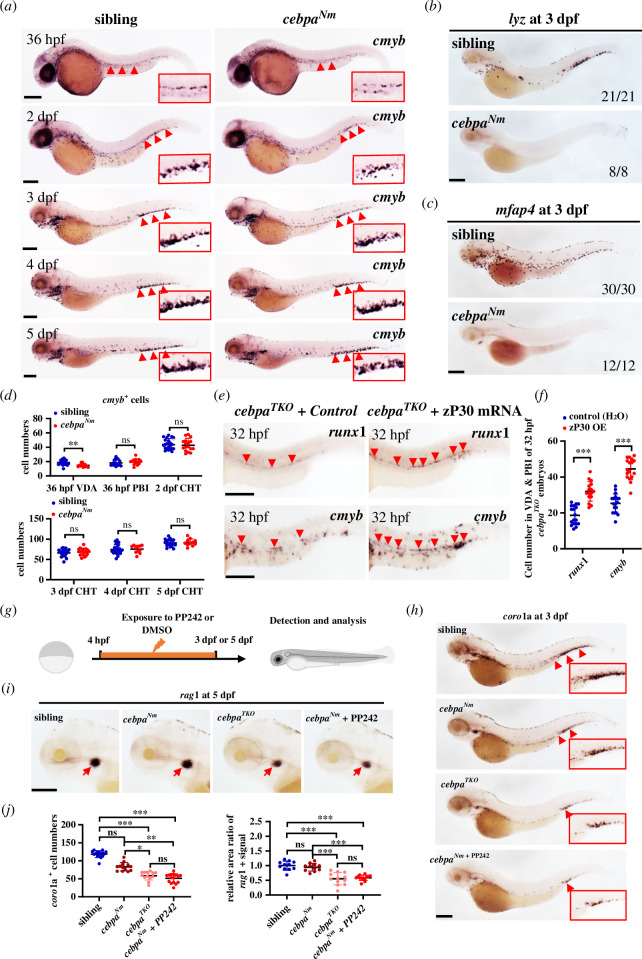
Truncated zP30 could compensate the role of Cebpa in regulating HSPC formation and multilineage development. (*a*) Comparable *cmyb*^+^ HSPCs presented in PBI or CHT between sibling and *cebpa^Nm^* mutants (zP30-expressing) from 36 hpf to 5 dpf by WISH, though a slight decrease of *cmyb*^+^ cells in the VDA was observed in mutants at 36 hpf. Red boxes showed enlarged signal details. (*b,c*) Absent *lyz* and *mfap4* expression in *cebpa^Nm^* mutants. WISH of *lyz* (*b*) and *mfap4* (*c*) in sibling and *cebpa^Nm^* embryos at 3 dpf. The numbers in the lower right corner indicated the ratio of embryos with representative expressing patterns in whole embryos with identical genotypes. (*d*) Quantification of *cmyb*^+^ cell numbers in the VDA, PBI or CHT at a specific stage of sibling and *cebpa^Nm^* mutants (mean ± s.d.; *n* ≥ 13; Student’s *t*‐test and non-parametric Mann–Whitney test, ns *p* > 0.05, **p < 0.01). (*e*) Forced zP30 overexpression increased *cmyb* and *runx1* expression in the VDA and PBI of *cebpa^TKO^* (*cebpa*-null) embryos. WISH of *runx1* (upper panel) and *cmyb* (lower panel) in *cebpa^TKO^* mutants with or without zP30 expression at 32 hpf. Red arrowheads showed *cmyb/runx1*^+^ signals in the tail. (*f*) Quantification of *runx1*^+^ or *cmyb*^+^ cells in the tails of control or zP30-expressing *cebpa^TKO^* embryos (mean ± s.d.; *n* ≥ 17; Student’s *t*‐test and non-parametric Mann–Whitney test, ***p < 0.001). (*g*) Flow chart of PP242 inhibitor treatment. Embryos were incubated with 5 μM PP242 (P30 translational inhibitory) or DMSO (control) from 4 hpf to destinated timepoint for further analysis. (*h,i*) Decreased *coro1a* (3 dpf) and *rag1* (5 dpf) expression in *cebpa^Nm^* zebrafish treated by PP242, similar to that in *cebpa^TKO^* mutants. WISH of *coro1a* (*h*) and *rag1* (*i*) in sibling, *cebpa^Nm^*, *cebpa^TKO^* embryos with DMSO or PP242 treatment. (*j*) Quantification of *coro1a*^+^ cell numbers (left) and relative *rag1*^+^ signal area (average signal area in sibling was considered as a reference) (right) in each group embryos related to (*h*) and (*i*) (mean ± s.d.; *n* ≥ 11; one-way ANOVA and Kruskal–Wallis test, ns *p* > 0.05, *p < 0.05, **p < 0.01, ***p < 0.001). Scale bars: 200 μm.

To determine whether HSPCs were affected in zebrafish with the *cebpa* N-terminal frameshift mutation, we monitored *cmyb* expression from 36 hpf to 5 dpf. Remarkably, though slightly decreased numbers in the VDA were exhibited in mutants prior to 2dpf, comparable *cmyb*^+^ cells were observed in the PBI or CHT of siblings and *cebpa^Nm^* mutant embryos at both early and later stages (figure 5*a*,*d*; electronic supplementary material, figure S7*e*,*f*), indicating almost unaffected HSPC generation and maintenance. Consistently, *rag1* expression at 9 dpf showed no significant differences (electronic supplementary material, figure S7*g*,*h*). However, myelocyte markers *lyz* and *mfap4* were completely absent in mutants at 3 dpf (figure 5*b,c*), indicating the same myeloid differentiation defect observed in other zebrafish *cebpa* mutants and P30-expressing mice [45,59,60].

There was evidence that shorter zP30 was synthesized by zebrafish *cebpa* with N-terminal frameshift mutations, such as the zD420 mutation, which mimics mammalian *CEBPA* N-terminal frameshift mutations to produce truncated proteins [52]. Therefore, we speculated that HSPC preservation in *cebpa^Nm^* mutants was due to functional zP30 production.

To test this hypothesis, we first assessed the translational re-initiation capacity of *cebpa^Nm^* mRNA through *in vitro* examination of GFP-tagged WT or *cebpa^Nm^* mutated Cebpa protein due to the lack of antibody in zebrafish. We found that compared with the full-length fusion Cebpa zP42 (approx. 65 kDa) encoded by WT mRNA, an approximately⌉10 kDa shorter isoform was detected in *cebpa^Nm^*-expressing cells (electronic supplementary material, figure S8*a*,*b*). Moreover, when GFP-tagged *cebpa^Nm^* mRNA was injected into *cebpa^TKO^* mutants, there was an increase in *cmyb*^+^ cells in the VDA and PBI regions of *cebpa^TKO^* embryos (electronic supplementary material, figure S8*c*–*e*). These data confirmed that the *cebpa^Nm^* mutation results in functional truncated zP30 expression but not zP42.

To further confirm the role of zP30 in the HSPC compartment, we synthesized zP30 mRNA and introduced it into *cebpa^TKO^* mutants. As expected, compared with mutant control, both HSPC markers *runx1* and *cmyb* were restored in zP30-expressing embryos at 32 hpf (figure 5*e*,*f*; electronic supplementary material, figure S8*f*). Additionally, *cebpa^Nm^* embryos showed disturbed generation of *cmyb*^+^ HSPCs when treated with PP242 (figure 5*g*; electronic supplementary material, figure S9*a*,*b*), which inhibits zP30 translation by blocking mTOR-mediated eIF4E activity [61,62]. We also examined the variation in *coro1a*^+^ myeloid progenitors and *rag1*^+^ lymphocytes in *cebpa^Nm^* embryos in response to zP30 deficiency, which resembled the complete loss of Cebpa observed in *cebpa*-null mutants. Results showed that PP242-treated *cebpa^Nm^* embryos displayed a consistent reduction in *coro1a* at 3 dpf and *rag1* at 5 dpf (figure 5*h–j*), comparable to *cebpa^TKO^* mutants, suggesting impaired differentiation potential of *cebpa^Nm^*-mutated HSPCs with zP30 loss. These observations lead us to conclude that Cebpa zP30 in zebrafish can sustain HSPC generation and subsequent multi-lineage differentiation in the absence of the full-length zP42.

### 3.6. *cebpa* acts downstream of *runx1* to participate in zebrafish HSPCs development

**Figure 6.**
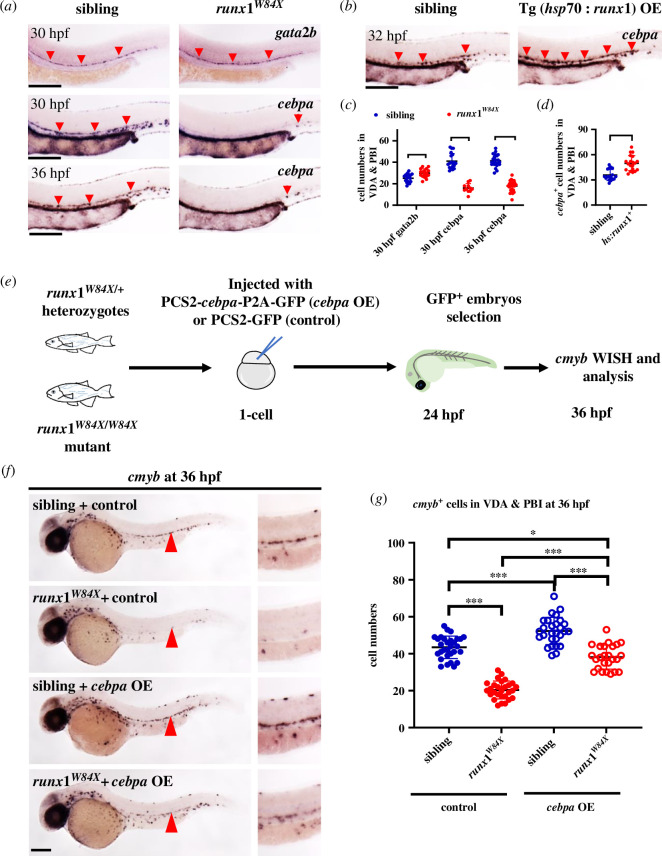
*cebpa* acted downstream of *runx1* in regulating HSPC development. (*a*) Severely decreased *cebpa* expression in *runx1*-deficient zebrafish at both 30 hpf and 36 hpf with increased *gata2b*^+^ cells. WISH of *gata2b* and *cebpa* in *runx1^W84X^* embryos and their sibling embryos at 30 hpf or 36 hpf. *gata2b*^+^ or *cebpa^+^* + were indicated by red arrowheads. (*b*) Elevated *cebpa* expression in *runx1*-overexpressing zebrafish as indicated by *cebpa* WISH in *Tg*(*hsp70:myc-runx1*)^+^ embryos and their sibling embryos at 32 hpf. (*c,d*) Quantification and comparison of *gata2b*^+^ or *cebpa*^+^ cells in the tails of each group (mean ± s.d.; *n* ≥ 11; Student’s *t*‐test and non-parametric Mann–Whitney test, ***p < 0.001). (*e*) Flow chart of rescue assay in *runx1* mutants by *cebpa* overexpression. PCS2-*cebpa*-P2A-GFP (*cebpa*-OE) or PCS2-GFP (control) vector was individually injected into one-cell staged *runx1* mutant or sibling embryos. GFP^+^ embryos were fixed at 36 hpf for *cmyb* WISH detection. (*f*) Reduced *cmyb*^+^ HSPCs in *runx1^W84X^* mutants was rescued by *cebpa* overexpression. WISH of *cmyb* in sibling or *runx1^W84X^* embryos with or without *cebpa* overexpression. Red triangles indicated enlarged signal details in the VDA (right panel). (*g*) Quantification of *cmyb*^+^ HSPCs in the tails of each group embryos (mean ± s.d.; *n* ≥ 26; one-way ANOVA, *p < 0.05, ***p < 0.001). Scale bars: 200 μm.

RUNX1 is a crucial regulator involved in multiple processes of HSPC development, including their generation and maintenance [10]. Previous research in mice has shown that RUNX1 induces *Cebpa* expression by directly binding to conserved promoter and enhancer regions, specifically in the context of myelopoiesis differentiation [63]. To investigate whether the RUNX1-CEBPA axis plays a role in HSPC formation, we examined *cebpa* expression in the VDA and PBI regions of embryos with *runx1* mutation during early embryogenesis. WISH revealed a drastic reduction of *cebpa* expression in the haematopoietic tissues of *runx1^W84X^* embryos, the *runx1*-deficient mutant with undiminished *gata2b*^+^ HE generation at 30 hpf [11,46], especially in the VDA where *cebpa* expression was nearly absent at both 30 hpf and 36 hpf (figure 6*a*,*c*). Conversely, in *Tg*(*hsp70:myc-runx1*) transgenic embryos with forced *runx1* expression induced by heat shock before HSPC emergence, *cebpa* RNA levels were markedly increased in the VDA (figure 6*b*,*d*). These data suggest that *cebpa* may be genetically regulated by Runx1 in nascent HSPCs.

To further elucidate the biological function of the RUNX1-CEBPA regulatory pathway in HSPC generation, we conducted rescue assays in *runx1* mutants by overexpressing *cebpa*. For continuous pan-overexpression of *cebpa* from early stages, we constructed a PCS2-*cebpa*-p2a-GFP plasmid and micro-injected it into one-cell stage embryos from out-crossed *runx1^W84X/+^* heterozygous and *runx1^W84X/W84X^* mutant adults (figure 6*e*). An examination of *cmyb*^+^ cells in GFP^+^ embryos at 36 hpf showed that *cebpa* restoration effectively recovered HSPC formation in *runx1*-deficient zebrafish. Additionally, *cmyb*^+^ HSPCs in the VDA were significantly increased in sibling embryos with exogenous *cebpa* expression (figure 6*f,g*). These results indicated that *cebpa* is indeed part of the downstream regulatory network of Runx1, governing HSPC emergence.

## 4. Discussion

This study highlights the significant role of zebrafish Cebpa in HSPC generation and maintenance during early embryogenesis. Utilizing endogenous *cebpa*-null and N-terminal frameshift zebrafish mutants, we observed severe impairment of HSPC formation and lymphopoiesis due to Cebpa deficiency, which could be rescued by zP30 production. Furthermore, we demonstrated that increased apoptotic cell death of HSPCs in the CHT was induced by Cebpa loss and that the RUNX1-CEBPA axis might be involved in HSPC modulation.

CEBPA is known to be widely expressed in various tissues and organs, including the liver, intestine, bone marrow and adipose tissue [64,65]. In haematopoiesis, CEBPA is predominantly expressed in myeloid progenitors and differentiated myelocytes [35], but it is also present in early HSPCs, primarily identified as LMPPs with both lymphoid and myeloid potential [32,51]. LMPPs are directly derived from HE cells through the EHT process in both mammals and zebrafish [3,66]. Our study provides *in vivo* evidence that *cebpa* is present in endothelium and nascent HSPCs in the VDA and PBI regions. Although co-localization experiments were only conducted at 36 hpf, we found that *cebpa^+^* cells were restricted to the VDA region at the earlier time points of 26 hpf and 30 hpf (electronic supplementary material, figure S1). Additionally, it has been reported that *cebpa* expression is enriched in the anterior- and posterior-lateral plate mesoderm (A- and P-LPM) between 12 hpf and 17 hpf, the regions where haematopoiesis is initiated in zebrafish [52]. Therefore, Cebpa may act at a higher hierarchical level in haematopoiesis. Total loss of Cebpa function in *cebpa^TKO^* mutants resulted in perturbed HSPC generation and preservation due to increased apoptosis. Subsequent myeloid and lymphoid lineage defects in definitive haematopoiesis were also detected. In addition to apoptotic cell death, decreased *cmyb/runx1^+^* nascent HSPCs may also result from obstacles of HE specification and the EHT process [1,67]. Our data showed unaffected expression of the HE-specific marker *gata2b* at 30 hpf in *cebpa*-null embryos (electronic supplementary material, figure S4*b*,*d*), suggesting that Cebpa is more likely involved in pre-HSC maturation and EHT transition to modulate HSPC generation in zebrafish and further direct evidence is required to confirm this hypothesis.

The requirement of CEBPA in HSPC generation has not been extensively studied, likely due to the difficulty of monitoring nascent HSPCs in mammals and *Cebpa*-null mutant die before birth. However, the increased apoptosis and reduced population of HSPCs observed in embryonic *cebpa^TKO^* zebrafish correspond to the behaviours of LT-HSCs in CEBPA knockout/knockdown mice [37,51]. HSPCs are the foundation for pre-leukaemic cell formation during leukemogenesis [68–70]. It was reported that intrinsic HSPC expansion caused by C-terminal bZIP in-frame mutated CEBPA^K313KK^, but not P30, accelerated AML progression in a mouse model with bi-allelic *Cebpa* mutations [36]. However, we found that HSPCs and definitive lymphocytes were unchanged in zebrafish with in-frame mutated *cebpa* in the bZIP domain (data not shown), suggesting that the HSPC exhaustion observed in bi-allelic *Cebpa*-mutated mice might result from the cooperative effect of two mutated CEBPA isoforms. Recent studies in mice clarified that HSPC depletion in CEBPA enhancer deletion mutants resulted from neutropenia due to continuous HSPC exhaustion by quiescence [51]. In our study, neutrophils were completely lost in all *cebpa*-mutated zebrafish, while HSPCs decreased only in *cebpa^TKO^* embryos, not in *cebpa^Nm^* and *cebpa^hkz7^* embryos, suggesting that HSPC defects were caused by Cebpa deficiency in a cell-autonomous manner. Further exploration is needed to determine whether Cebpa acts through its own transcriptional regulation or through interactions with other factors in the HSPC compartment.

Interestingly, in our study, *cmyb*^+^ HSPCs in the VDA region of *cebpa^Nm^* embryos before 2 dpf showed a slight decrease compared to control embryos (electronic supplementary material, figure S7*e*,*f*). The overexpression of zP30 mRNA in *cebpa^TKO^* mutants could not fully rescue HSPC numbers in the VDA region but more efficiently restored *cmyb/runx1*^+^ cells in the PBI (electronic supplementary material, figure S8*f*). High-fidelity single-cell tracing in zebrafish identified haematopoietic progenitors distinct from HSCs originating directly from HE cells in both the VDA and PBI regions, with lymphoid or myeloid potential [3]. This concept was further validated in mice, showing that HSCs and multipotent progenitors independently derive from HE cells [66]. Subsequent research indicated that HSC-independent embryonic progenitors contribute more significantly to early haematopoiesis, especially lymphocyte and myelocyte generation before 8 dpf in zebrafish [71]. Indeed, *rag1*^+^ T-cells significantly decreased in *cebpa^TKO^* mutants from 5 dpf to 9 dpf. It remains unknown whether lymphocytes are also disturbed at later stages beyond 15 dpf in zP30-expressing *cebpa^Nm^* zebrafish, as *rag1* levels were unaffected at 9 dpf. Investigating whether Cebpa zP42 or zP30 isoforms are distinctly involved in HSC or HSC-independent progenitor emergence will be meaningful.

As reported, *cebpa^hkz7^* mutants harbour a C-terminal frameshift mutation within bZIP domain, leading to dysfunctional Cebpa protein synthesis [45]. Here, we found that *cmyb^+^* + and the lymphoid population developed normally in *cebpa^hkz7^* mutant embryos. Otherwise, in other publications of Zhu’s team, they claimed that Cebpa showed a negative impact on HSPCs maintenance [72,73]. These conclusions are overturned by our novel *cebpa*-null mutant showing severe HSPC defects. The phenotypic diversity might be explained by the different mutation alleles presented in the two zebrafish lines. Compared to truncated Cebpa protein expression, the mutant with an 820 bp deletion covering translational initiation sites and nearly the entire Cebpa coding exon more persuasively abolishes Cebpa protein function. It is well known that bZIP-mediated dimerization and DNA-binding abilities are indispensable for Cebpa’s transcriptional activation. Therefore, we postulate that the interaction of Cebpa with other factors outside bZIP might play a key role in HSPC regulation. Further study is required to dissect the molecular mechanisms of Cebpa in HSPC development.

In summary, our work indicates a novel role of *cebpa* in HSPC generation and confirms that Cebpa acted protectively in maintaining the HSPC pool through targeted genetic *cebpa* interference. We also show that truncated Cebpa zP30 exhibits a compensatory effect in the HSPC compartment, providing valuable insights into HSPC regulation by Cebpa during early embryogenesis.

## Data Availability

Supplementary material is available online [74].
